# Application of Water Hyacinth Biomass (*Eichhornia crassipes*) as an Adsorbent for Methylene Blue Dye from Aqueous Medium: Kinetic and Isothermal Study

**DOI:** 10.3390/polym14132732

**Published:** 2022-07-04

**Authors:** Marcelo T. Carneiro, Ana Z. B. Barros, Alan I. S. Morais, André L. F. Carvalho Melo, Roosevelt D. S. Bezerra, Josy A. Osajima, Edson C. Silva-Filho

**Affiliations:** 1 Federal Institute of Piauí, Floriano Campus, Floriano 64808-475, PI, Brazil; marcelo.teixeira@ifpi.edu.br (M.T.C.); andreluiz@ifpi.edu.br (A.L.F.C.M.); 2 Interdisciplinary Laboratory for Advanced Materials, Teresina 64049-550, PI, Brazil; anazuleica2@gmail.com (A.Z.B.B.); alanicaro@gmail.com (A.I.S.M.); josyosajima@ufpi.edu.br (J.A.O.); 3 Federal Institute of Piauí, Teresina-Central Campus, Teresina 64000-040, PI, Brazil; rooseveltdsb@ifpi.edu.br

**Keywords:** water hyacinth, *Eichhornia crassipes*, bioadsorbent, removal, methylene blue

## Abstract

Water pollution has generated the need to develop technologies to remove industrial pollutants. Adsorption has been recognized as one of the most effective techniques for effluent remediation. In this study, parts (stem and leaves) of a problematic aquatic weed, the water hyacinth (*Eichhornia crassipes*), were separated to produce a bioadsorbent. The objective was to evaluate the adsorption of a cationic dye, methylene blue (MB), in an aqueous solution of the biomass from different parts of the water hyacinth (*Eichhornia crassipes*) plants. The materials were characterized through techniques of infrared spectroscopy, scanning electron microscopy, X-ray diffractometry, and thermogravimetric analysis, before and after the material adsorption. Water hyacinth biomasses presented adsorption capacity above 89%, and the kinetics was faster for stem biomass. The kinetic study found that the adsorption process is better described by the pseudo-second-order model, and the adjustments of the isotherm experimental data indicated that both materials are favorable for adsorption. Therefore, water hyacinth bioadsorbent represents a renewable resource with potential for effluent treatment.

## 1. Introduction

One of the consequences of social growth and development is the generation of large amounts of residues from different synthetic compounds. The considerable contamination of effluents has become a severe environmental problem and a serious threat to fauna and flora, and human beings [1,2]. In recent decades, many industries such as weaving, dyeing, papermaking, leather, pharmaceuticals, and chemical production used a significant number of dyes in their manufacturing process, leading to the release of hazardous effluents into drinking water [3], which have caused serious environmental problems [4].

Contamination by new emerging contaminants such as drugs and pharmaceutical residues [5], heavy metals, textile dyes, pesticides, and surfactants [6] is a major concern, mainly due to the negative consequences for human and aquatic ecosystem health [7,8]. Many emerging effluents are proven to cause effects at a biochemical level due to their toxicity, carcinogenicity, and mutagenicity [9]. It is estimated that at least 100,000 of these compounds are produced regularly, equivalent to 200 million tons per year, of which 20–30% can contaminate aquatic environments [10].

For this reason, immediate removal of the methylene blue (MB)dye from water using appropriate techniques is necessary to protect humans from adverse health effects [11], mainly due to the limited accessibility of high-quality clean water sources [11,12].

Adsorption has been shown to be one of the most efficient processes due to its flexibility, efficiency, low cost, and reversibility [6,13].

Research has shown the application of different adsorbents, such as activated carbons [10,14], nanoadsorbents [2,5], clays [15], and cellulosic derivatives [6,16], for the removal of pollutants present in the aquatic environment, have obtained varied adsorption efficiencies. The use of bioadsorbents–adsorbent materials of natural origin–for the treatment of effluents is a promising proposal due to their greater adsorption capacities, the economic aspects (low cost), and their availability in mass [17].

Among the raw materials that can be used to produce bioadsorbents, is water hyacinth (*Eichhornia crassipes*). This plant has high proliferation in eutrophic water environments [18] and is globally recognized as an invasive species that threatens the survival of aquatic organisms by blocking sunlight and, thus, depriving aquatic life of environmental resources such as atmospheric oxygen [19].

Therefore, Water hyacinth residue is a possibility to consider for the synthesis of new adsorbents in the removal of environmental contaminants, in addition to adding commercial value to the product

This work aimed to analyze the efficiency of MB textile dye removal in aqueous solutions through the adsorption process using water hyacinth (*Eichhornia Crassipes*) biomass as bioadsorbent. To verify a useful destination for the expressive amount of this plant, two bioadsorbents were produced (leaf and stem of the water hyacinth) that were characterized by Fourier-transform infrared spectroscopy (FTIR), scanning electron microscopy (SEM), X-ray diffractometry (EDX), and thermogravimetric analysis (TG). The compositions of the fibrous fractions were determined and the adsorption tests were performed by varying the pH, time, and concentration of the dye. The experimental tests were adjusted to different kinetic and isothermal models. Finally, the desorption and reuse processes of the material were carried out. 

## 2. Materials and Methods

### 2.1. Materials and Reagents

Methylene blue (C_16_H_18_SN_3_Cl, Dinâmica–Company originating in the country itself - Brazil), Sodium hydroxide (NaOH, Dynamic, 98%), Hydrochloric acid (HCl, Dynamic, 38%), Sodium chloride (NaCl, Dynamic, 99%), and Distilled water were provided by the Interdisciplinary Laboratory of Advanced Materials (LIMAV) of the Federal University of Piauí (UFPI), Teresina, Piauí, Brazil and the company Dinâmica Química Contemporânea LTDA, Indaiatuba, Brazil with more than 30 years in the market, certified with ISO 9001:2015. All reagents were analytical grade, and no prior purification was required. The water hyacinth (*Eichhornia crassipes*) was obtained from the Matias Augusto Oliveira Matos Environmental Park, in the city of Teresina (PI), Brazil.

### 2.2. Preparation of Adsorbent Material

The roots of the water hyacinth plants were removed and discarded, and the plants were washed in running water and left to dry for three days in the open. Then, the stems and leaves were separated and placed in an oven at 50 °C for 24 h [20]. After the drying process, they were ground separately and left in an oven at 45 °C for 24 h, and finally, sieved using a 425 µm particle size sieve.

### 2.3. Biomass Characterization

The Fourier-transform Infrared Spectroscopy (FTIR) technique was used to determine which functional groups are present in biomass. The spectra were obtained using a Bruker spectrophotometer, model Vertex 70, in the ATR configuration. Spectra were obtained with 60 scans in the range of 600 to 4000 cm^−1^. The morphology of the materials prepared from water hyacinth was obtained by scanning electron microscopy (SEM), using a scanning electron microscope model MEV Tescan® Vega3® LMU®, Sâo Bernardo do Campo, Brazil, at a voltage acceleration of 30 kv at magnifications of 100× and 500×, to explain the shape and surface properties of the adsorbent. In addition, X-ray diffraction (XRD) was used to observe the crystallinity of the biomass before and after adsorption. The analysis was performed in an X-ray diffractometer (Labx-XRD 600, Shimadzu, Nakagyo-ku, Kyoto, Japan), using Kα-Cu radiation = 1.5418 Å, in a 2θ range from 5° to 75°, acceleration voltage and applied current of 40 kV and 30 mA, respectively. Thermal stability was evaluated by thermogravimetric analysis (TGA) in a thermal analyzer (DT-60, Shimatzu, Nakagyo-ku, Kyoto, Japan) using a heating rate of 10 °C/min between 25 and 600 °C, in an inert nitrogen atmosphere.

The composition of the fibrous fraction of the stem and leaf of the water hyacinth (*Eichhornia crassipes*) was performed to determine first the dry matter (DM; method 967.03) and then the fiber fractions (in triplicate), according to the recommendations of the Association of Official Analytical Chemists [21]. Neutral detergent fiber (NDF) and acid detergent fiber (ADF) were determined (triplicate) according to the methodology proposed by Van Soest et al. [22]. Acid detergent lignin (ADL) was obtained from ADF residue incinerated in an oven at 600 °C for 4 h according to the methodology described by Van Soest [23]. Cellulose and hemicellulose were calculated from the following proposal: Hemicellulose = NDF − ADF and Cellulose = ADF − ADL [23].

### 2.4. Point of Zero Charge (pH_pzc_)

The methodology consists of determining the pH at which there is a balance of charges between the surface of the adsorbent and the solution [24]. For this, 20.00-mg samples of biomass from stems or leaves were added to 20.00 mL of NaCl (0.1 mol∙L^−1^), where the pH was adjusted to correspond to the range from 2 to 12 [6,16]. The pH was adjusted with two solutions of HCl, one of 1.0 mol∙L^−1^ and one of 0.1 mol∙L^−1^, as well as two solutions of NaOH at the same concentrations. The pH reading was performed using a pH meter [16,25]. The mixtures were left under constant stirring at 140 rpm for 24 h at 25 °C on a shaker table (model TE-420 INCUBATOR - TECNAL). After the contact time, the solutions were centrifuged (model NI 1812 Benchtop Centrifuge - INOVA Instruments) for 10 min at 5000 rpm or 4980 rcf, and then the final pH of the solution was measured. The difference between the initial and final pH was plotted from the data obtained using Equation (1), identifying the intersection where ΔpH = 0 corresponds to the point of zero charge, which is pH_pzc_ [25,26].

(1) $$\Delta \mathrm{pH}={\;\mathrm{pH}}_{0}-{\;\mathrm{pH}}_{f}$$

### 2.5. Influence of pH

The effect of pH on dye adsorption was studied at pH 1, 3, 5, 7, 9, and 11, where the pH was adjusted with NaOH and HCl solutions. Maintaining the concentration of 300.00 mg∙L^−1^ of the dye, the study was carried out in triplicate at 25 °C [25,27]. The 100.00 mg samples of biomaterials were placed in contact with 20 mL of MB 300.00 mol∙L^−1^ solution. The system was agitated for 48 h. After the contact time, the solutions were centrifuged and diluted. The new concentrations were determined by reading the aliquots in a UV-Vis spectrophotometer (Agilent, Santa Clara, CA, USA, Cary 60) using a pre-established calibration curve.

Finally, the adsorbed amount Qe (mg∙g^−1^) and the adsorption efficiency were calculated according to Equations (2) and (3), respectively [25,28,29].

(2) $$\mathrm{Qe}=\frac{\left(C_{i}-{\;C}_{f}\right)}{m}\;V$$

(3) $$R=\frac{\left(C_{i}-{\;C}_{f}\right)}{C_{i}}100\%$$

where C_i_ and C_f_ represent the initial and final concentrations (mol∙L^−1^) of the dye, respectively; m corresponds to the mass of the adsorbent in grams, and V equals the volume in liters of the dye solution used.

### 2.6. Adsorption Kinetics

The kinetic adsorption tests were conducted in triplicate, at a temperature of 25 °C and natural pH of the dye solution (7.01). Initially, 100 mg of leaf or stem biomass were placed in contact with 20.0 mL of MB solution and kept under constant stirring at 140 rpm for 4 h [6,25]. After the contact time, aliquots were centrifuged and determined in a UV-Vis spectrophotometer using the preestablished calibration curve. Finally, the obtained kinetics were suitable for pseudo-first-order and pseudo-second-order kinetic models.

The linearized form of the pseudo-first-order [30] and pseudo-second-order [31] kinetic models are expressed in Equations (4) and (5), respectively.

(4) $$\ln\;\left(q_{e}-{\;q}_{t}\right)={\ln\;q}_{e}-{\;K}_{1}.t$$

(5) $$\frac{t}{q_{t}}=\frac{1}{k_{2}\Delta {\;q}_{e}^{2}}+\frac{t}{q_{e}}$$

where q_e_ and q_t_ are the amounts of dye adsorbed (mg∙g^−1^) at equilibrium and at time t, respectively; K_1_ represents the adsorption constant of the first-order model (min^−1^) and K_2_ is the constant of the pseudo-second-order kinetic model (mg∙g^−1^ min^−1^) [6,25].

### 2.7. Adsorption Isotherms

The test was carried out in triplicate at 25 °C, with pH adjusted according to the best adsorption, in which 100 mg of biosorbent was added to 20 mL of dye in solution, varying the concentrations between 50 and 1000 mg∙L^−1^, which were stirred in the best equilibrium time found in the kinetics experiment [6]. After centrifuging the samples, measurements were made in a UV–Vis spectrophotometer to calculate the equilibrium concentrations resulting from the adsorption of each initial dye mass. This was to investigate which adsorption isotherm model the experimental data fit best (Langmuir or Freundlich).

Equation (6) expresses the linearized form of the Langmuir equation [32]:

(6) $$\frac{C_{\mathrm{eq}}}{q_{e}}=\frac{1}{q_{\max}}.{\;C}_{e}+\frac{1}{K_{L}.q_{\max}}$$

where q_e_ is the amount of adsorbed species per mass of the bioadsorbent (mg∙g^−1^), q_max_ is the maximum amount of adsorbed species per mass of the bioadsorbent (mg∙g^−^^1^), C_eq_ represents the equilibrium concentration of the adsorbent (mg∙L^−1^), and K_L_ is the Langmuir adsorption constant related to the chemical balance between adsorbent and adsorbent (mg∙L^−1^) [33]. 

The linearized form of the Freundlich equation can be observed by Equation (7) [34].

(7) $$\ln q_{e}=\ln K_{F}+\frac{1}{n}\ln C_{e}$$

where K_F_ is the Freundlich adsorption constant related to the adsorption capacity, 1/n is the constant related to the surface heterogeneity, q_e_ represents the number of species adsorbed per mass of the bioadsorbent (mg∙g^−1^), and C_e_ represents the concentration equilibrium of the adsorbate (mg∙L^−1^) [25].

### 2.8. Material Reuse

The reuse of stem and leaf in the adsorption process was conducted three times: adsorption, desorption, and reuse. At the first adsorption time, 0.5 g of the biomass was placed in contact with 100 mL of methylene blue dye at a concentration of 600 mg∙L^−1^ and left under stirring at 130 rpm for 60 min. Aliquots were removed and centrifuged at 14,000 rpm for 35 s to separate the solid from the supernatant. The second time, the biomass powders were recovered by filtration with filter paper and washed with methanol to remove the dye adsorbed on the materials and dried at 40 °C for 12 h. The reuse of the material was carried out two more times, respecting the entire process described above. With the UV-Vis spectrophotometer, using the calibration curve, aliquots were analyzed in each reuse process [35,36].

## 3. Results

### 3.1. Characterization of Water Hyacinth Biomass

**Figure 1:**
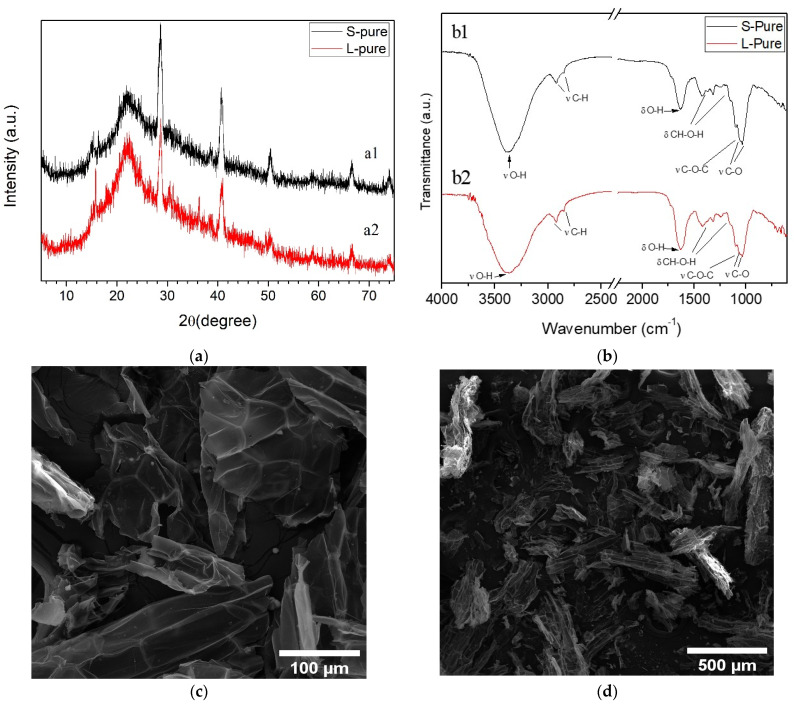

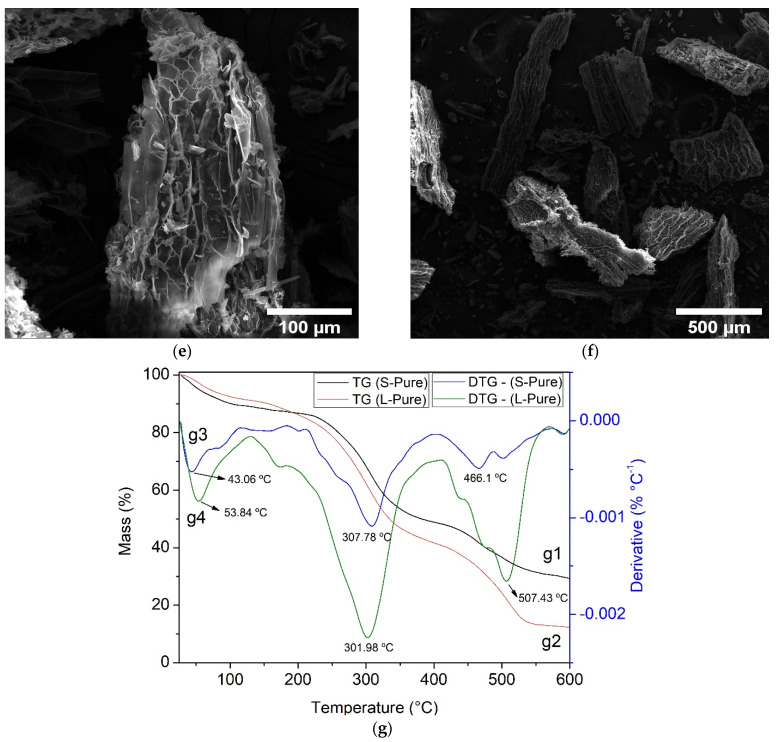
(**a**) X-ray diffractograms of the water hyacinth biomass: (a1) stem and (a2) leaf of; (**b**) FTIR spectra of (b1) stem and (b2) leaf; SEM—micrograph of the water hyacinth parts: (**c**,**d**) stem; (**e**,**f**) leaf. (**g**) TG thermogravimetric curves of water hyacinth (g1) stem and (g2) leaf. DTG: water hyacinth (g3) stem and (g4) leaf.

Figure 1a is the X-ray diffractogram for the studied biomasses. This analysis was used to study changes in the crystalline structure of biomass, where the peaks in the graph indicate the crystallinity of the material.

Cellulose and hemicellulose constitute about 65% to 70% of lignocellulosic biomass, including water hyacinth [37]. Cellulose is highly ordered, with 80% of crystalline regions. In Figure 1 (a1 and a2), the characteristic peaks at approximately 2θ = 15.50° and 22.12° indicate the crystallinity of water hyacinth biomass [38,39]. The semi-crystalline profile of the water hyacinth is due to the lignin in its structure because the lignin is amorphous, while cellulose is predominantly crystalline [40]. Other peaks observed may refer to impurities present in the material, due to the capacity of this plant (water hyacinth) to remove inorganic substances from water bodies, such as heavy metals [37]. 

The FTIR spectra of the water hyacinth parts are shown in Figure 1b and display bands characteristic of cellulose. The cell wall evidently consists mainly of lignin, hemicellulose, and cellulose (among other elements in a smaller proportion), providing functional groups such as amines, carboxyls, hydroxyls, sulfhydryl, carbonyls, involved in the removal of pollutants from water [41].

In the range of 3300 cm^−1^, the band refers to the stretch vibrations v(O-H) present in the ring and in the side chain [42]. The bands, corresponding to the stretching of the methylene groups v(C-H), (CH_2_ and CH_3_), are located in the region of 3000–2800 cm^−1^ [26]. The bands attributed to the symmetric angular deformations δ (CH_2_) and vibrations of asymmetric elongation ν(C-O-C) of the glycosidic bonds and elongation ν(C-O) are observed at 1423 and 1372 cm^−1^, 1160, 1054, and 1113 cm^−1^, respectively [6,43,44]. The band at 1249 cm^−1^ indicates C-O elongation in lignin and hemicellulose [44,45]. The band at 1700 cm^−1^ either represents acetyl or uronic ether bonds of carboxylic groups in ferulic acids and p-coumaric acids, as both acids can be found in lignin [40].

Figure 1 shows the morphological structures of the stem (c and d) and leaf (e and f) of the water hyacinth at 500× and 100× magnifications, to explain the shape and surface properties of the adsorbent. Figure 1c and d refers to parts of the stem and displays fibrous rods with an irregular surface structure with cavities. Figure 1e,f, referring to part of the water hyacinth leaf, shows nonhomogeneous structures with some cavities, thus justifying the need to carry out studies with separate parts, since morphology and porosity can influence the adsorption capacity. The presence of an irregular surface, pores, and surface fractures of both the water hyacinth leaf and stem indicate the presence of micropores and a high surface area [46], which are fundamental and important characteristics of adsorbents [47]. Since these small spaces favor the entry of liquids and adsorption [48].

The analysis of its biosorbent micrograph indicates that the stem in this natural condition has greater adsorption potential. This was also identified in other studies involving aquatic macrophytes of different species that exhibit heterogeneous morphology, with porous with an irregular rough surface full of cavities that provide a perfect state of adsorption [49,50]. 

The thermogravimetric curves (TG) of the materials are shown in Figure 1 (g1 and g2) for the stem and leaf, respectively. The derivative of thermogravimetric curves (DTG) is in Figure 1 (g3 and g4) for the stem and leaf, respectively. TG and DTG in Figure 1 (g1 and g3) show that the stem thermal profile can be divided into three stages. The first stage below 260 °C occurs at around 43 °C maximum temperature and is related to loss of adsorbed water, some dehydration of the cellulose structural units, and loss of volatile compounds. The second stage between 260 and 400 °C, in which the maximum temperature event occurs at 307 °C, is probably the thermal decomposition of the material [48], hemicellulose and the β-(1→4)-glycosidic bonds of cellulose. The third stage, above 400 °C, with a maximum temperature of 466 °C, probably involves the decomposition of the remaining cellulose and lignin [51]. 

Data for leaf material, Figure 1 (g2 and g4) for TG and DTG, respectively, indicate a similar three-stage stem thermal profile, but with changes in maximum temperature events. The first stage had a small increase in the maximum temperature of the event to 53 °C, the second stage had a slight decrease to 301 °C, and in the third stage, the maximum temperature of the event occurred at 506 °C [52]. Observing the residual mass of the materials at 600 °C (Figure 1 g1 and g2), the stem showed a residual content higher than the leaf with 29% and 12%, respectively. A reason for this change may be related to the slight difference in the concentration of hemicellulose, lignin, and mineral components in greater quantity in the stem than in the leaf, and this may also be one of the factors for the ash content formed [53].

Table 1 presents the mean and standard deviation of the composition of the fibrous fractions of the stem and leaf of the water hyacinth. The water hyacinth stem had a higher proportion of lignin of 10.88% compared to 8.36% for leaf, as well as cellulose (27.79% for stem and 20.81% for leaf) and hemicellulose (30.24% for stem and 29.71 % for leaf) indicating that the leaf can biodegrade faster than the stem [54].

The composition of water hyacinth appropriately corresponds to the standard composition of wet aquatic biomass. According to the literature, it consists mainly of cellulose and hemicellulose and generally has a low lignin content [55], constituting lignocellulose which is hygroscopic and has an affinity for water, being able to permeate the noncrystalline portion of water [56].

Water hyacinth biomass (both leaf and stem) in the form of bioadsorbent is more relevant, as it contains cellulose, hemicellulose, and lignin in proportional amounts compared to other mostly acquired biomasses, such as rice straw, wood chips, and sugarcane bagasse [57]. The constituents mentioned above contain the different functional groups, such as carboxylate, aromatic carboxylate, oxyl, and phenolic hydroxyl groups, which have good potential for adsorption [58]. The existence of numerous hydroxyl groups in the cellulose backbone of this aquatic plant is primarily responsible for adsorption, in addition to having the potential to be chemically modified to improve the adsorption process [19].

The pH is a fundamental parameter in adsorption since it indicates the degree of distribution of loads in the solution [59]. The point of zero charge (pH_pzc_) determines an index at which a surface tends to become positively or negatively charged as a function of pH. The adsorption mechanism of organic molecules from dilute aqueous solutions in carbonaceous materials is known to be a complex interaction between electrostatic, non-electrostatic, and hydrophobic interactions. These interactions depend on the characteristics of the adsorbent and the adsorbate, thus emphasizing the importance of pH_pzc_ [60]. 

**Figure 2:**
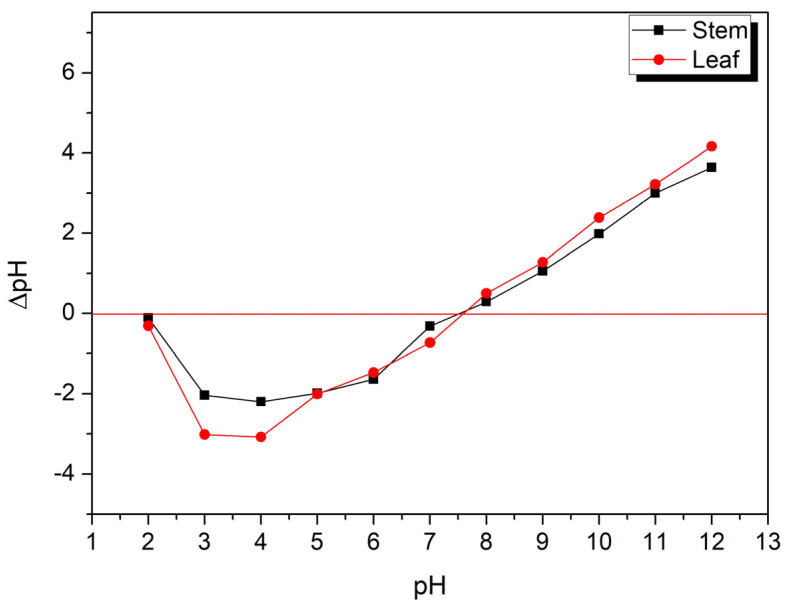
Representation of pH_pzc_ for water hyacinth stem and leaf materials.

Figure 2 illustrates the ΔpH values of the materials, where the points that intercept ΔpH = 0 correspond to the pH_pzc_. The estimated pH_pzc_ values were approximately 7.4 and 7.6 for the stem and leaf samples, respectively. For the pH values below pH_pzc_, the surface charge is positive indicating that there are favored adsorption of anionic species, while values above pH_pzc_ favor adsorption of cationic species [59,60].

### 3.2. Adsorption Experiments

**Figure 3:**
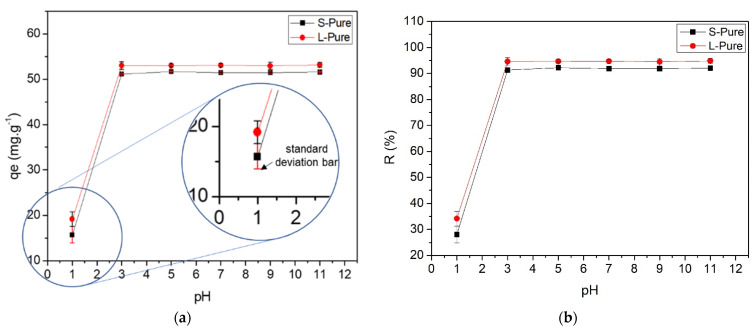
(**a**) Relation between the amount of dye adsorbed as a function of pH and (**b**) adsorption efficiency.

The behavior of the adsorbed amount as a function of pH was studied by varying the pH of the MB solution. As illustrated in Figure 3, this variable is very important in its ability to remove the dye. The obtained results showed that the most effective removal occurred in the range of pH 3 to 11, both for the part of the stem and for the leaf of the water hyacinth.

Other lignocellulosic materials report that the adsorption of MB by the water hyacinth plant is significantly improved in solutions where the pH is greater than 2 [46]. The high pH may increase the number of negatively charged binding sites, facilitating the removal of MB by dry biomass surfaces [61]. This can be due to the protonation of carboxyl and hydroxyl groups present on the surface of the material (water hyacinth) at a pH lower than pH_pzc_ (Figure 2), leading to an increase in positive charges that repel MB cations. Considering that the pKa of the MB is 3.8, above this pH value, the cationic species were the preponderant MB species in the solutions [62]. This behavior as a function of pH was also shown in MB adsorption studies in other lignocellulosic materials [63]. 

Finally, for both the S-Pure and L-Pure material, the amount of adsorbed dye, from pH 3 to 11, was statistically equal, around 91.87% ± 0.17 for stem and 94.68% ± 0.01 for leaf (Figure 3). As the adsorption was favorable and had little variation from pH 3 values, we chose to use the natural pH (without pH control) of the MB solution (7.01) for the following tests, because the process is easier to optimize, without the need for pH control, reducing costs.

It is evident that the water hyacinth as a raw material for the constitution of bioadsorbents has natural advantages, such as a spongy structure with large cellular gaps, which can generate a significant specific surface area. In general, the greater the specific surface area of the biomass, the greater the pore volume, which can provide greater adsorption capacity [64]. Its surface presents a slightly more basic character, indicating that it can adsorb cationic dyes more easily in a certain pH range, but it also includes some acidic characteristics, indicating the presence of different active sites on its surface. This can be explained by the fact that S-Pure and L-Pure have adsorption capacities at pHs 3 and 5 [65].

**Figure 4:**
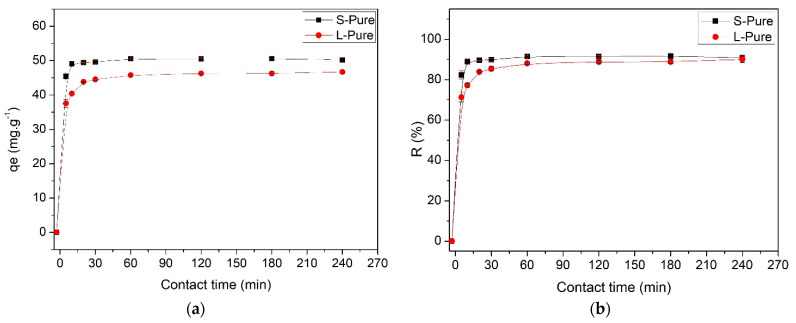
(**a**) Time isotherm for MB removal by water hyacinth stem and leaf biomass and (**b**) adsorption efficiency.

Starting from solutions with a concentration of 300.0 mg∙L^−1^ allows observation of the adsorption kinetics of the system, in which the dye removal rates were fast during the initial contact time. Stem and leaf biomass removed approximately 50.42 mg∙g^−1^ ± 0.15 (90.8%) and 46.88 mg∙g^−1^ ± 0.25 (89.9%) of the total adsorbate, respectively. The amount adsorbed increased rapidly over time, until the system reached equilibrium in approximately 60 min, after which there was no significant change in concentration, both for stem and leaf, indicating a rapid interaction, which is an extremely favorable parameter (Figure 4).

**Figure 5:**
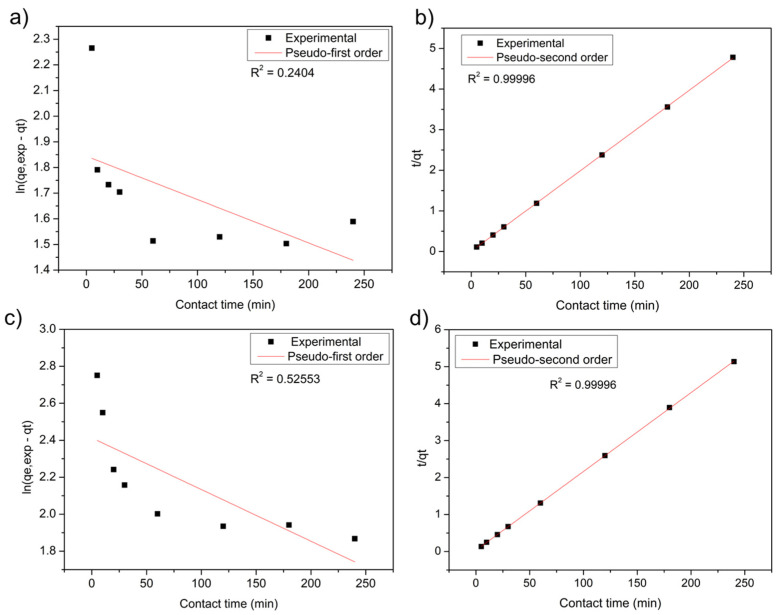
Adjustment to the kinetic model for the stem: (**a**) pseudo-first- and (**b**) pseudo-second-order for the MB in contact with the biomass. For the leaf: (**c**) pseudo-first- and (**d**) pseudo-second-order for the MB in contact with the biomass.

From the data obtained (Figure 4), the adjustments of the pseudo-first-order and pseudo-second-order models were calculated to observe which is the most appropriate to describe the mechanism of MB adsorption in the studied biomaterials (Figure 5). The calculated kinetic parameters are shown in Table 2.

Figure 5 demonstrates that the experimental data were adjusted to the pseudo-second-order model, both for the systems using the leaf adsorbent and the stem, which obtained linearity coefficients R^2^ = 0.99996 (Figure 5b,d). This model assumes that the rate-determining step depends on the physicochemical interactions between the adsorbate and the adsorbent surface groups, thus indicating a chemisorption process [66,67].

The analysis can also be interpreted when comparing the amount of adsorbed dye (q_e_) and the reaction rate constant (K) of the stem and leaf biomasses by pseudo-first- and second-order models (Table 2) [68]. The pseudo-second-order values present better results than the pseudo-first-order.

Several authors found results adjusted to the pseudo-second-order model using bioadsorbents. For example, Jahangiri et al. [67] worked on Pb(II) lead removal from aqueous solutions and wastewater using water hyacinth (*Eichhornia crassipes*) roots. The kinetic data indicated that Pb(II) adsorption followed the pseudo-second-order model with a reaction rate constant (K) of 0.0127 (mg.g^−1^.min^−1^). The kinetics data for the pseudo-second-order model suggest that chemisorptions were the rate-limiting step in the adsorption process. The research by Prasad et al. [46] found that *Eichhornia crassipes* is an excellent biosorbent for industrial effluent treatment. The authors also observed the adsorption kinetic with a correlation coefficient R^2^ = 0.99, very close to 1, showing that the pseudo-second-order model was better than the pseudo-first-order model.

The adsorption isotherm represents the equilibrium relationship between the amount of material adsorbed and the concentration in the fluid phase at a constant temperature. Many isotherm equations exist to adjust the experimental data, and among the most used are the equations proposed by Langmuir and Freundlich, due to their ability to predict the behavior of experimental data [32,34]. 

**Figure 6:**
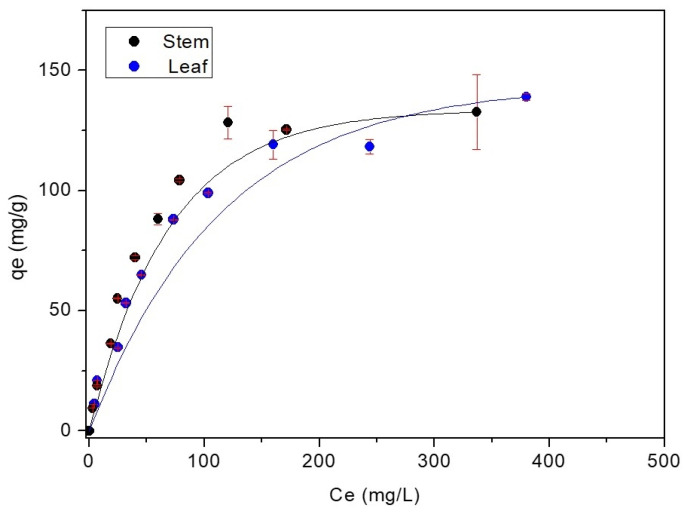
MB adsorption isotherm for water hyacinth stem and leaf biomass.

Plotting the values obtained, the experimental isotherms of the dye are shown in Figure 6.

The downward concave adsorption isotherm indicates a strongly favorable isotherm that leads to greater adsorption capacity [69]. When analyzing the adsorption isotherm in Figure 6, the plotted curves illustrate that the adsorption for both stem and water hyacinth leaf biomass is favorable, indicating that the adsorption process occurs even at low concentrations.

**Figure 7:**
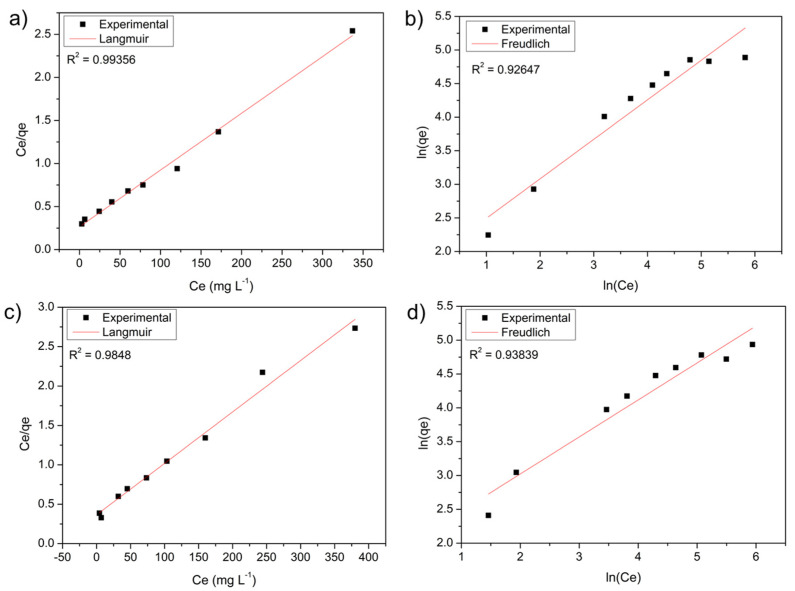
Linearized isotherm of (**a**) Langmuir and (**b**) Freundlich for the adsorption of stem biomass; linearized concentration isotherm of (**c**) Langmuir and (**d**) Freundlich for leaf biomass adsorption.

By linearizing the obtained data, it was possible to adjust the experimental data to the Langmuir and Freundlich models for stem (Figure 7a,b) and leaf (Figure 7c,d) biosorbents. The isothermal parameters are in Table 3.

The data fit better the Langmuir model, with R^2^ linearity value closer to 1 for both stem and leaf of the water hyacinth, as shown in Figure 7a,c, respectively. This model assumes that the solid surface is homogeneous and restricted to monolayers, characterizing a fixed number of active binding sites with the same affinity as MB. However, the experimental data has a good fit for the Freundlich model (Figure 7b,d), which considers interactions on heterogeneous multilayer surfaces with different affinities and interaction energies between MB and the active sites on the surface of the stem and leaf. This model also predicts that the adsorption process is favorable, as the R² linearity value was close to 1 for both the stem and leaf [70]. The Langmuir model was observed in other adsorption studies using water hyacinth biomass [71].

Corroborating our results, Kulkarni et al. [72] performed isothermal tests using water hyacinth root powder to remove Congo red dye in batch operation and in a continuous packed bed, whose values described the best result for Langmuir, where the maximum adoption capacity was 93.82 mg.g^−1^.

Jahangiri et al. [67] performed isothermal tests using water hyacinth (*Eichhornia crassipes*) roots to remove lead from aqueous solutions and wastewater. The values described the best result for Langmuir (R^2^ = 0.986), and the maximum adsorption capacity was 50 mg.g^−1^ Pb(II) of dry roots.

Observing the results in Table 3, the maximum adsorption capacity of MB was approximately 153.84 mg.g^−1^ for stem and leaf biomass in a solution of an initial concentration of 1000 mg∙L^−1^, which demonstrates the potential of stem and leaf to remove this dye in an aqueous medium. The value of the Freundlich constant was not evaluated, because the Langmuir data were better.

### 3.3. Characterization of Water Hyacinth Biomass after Adsorption

**Figure 8:**
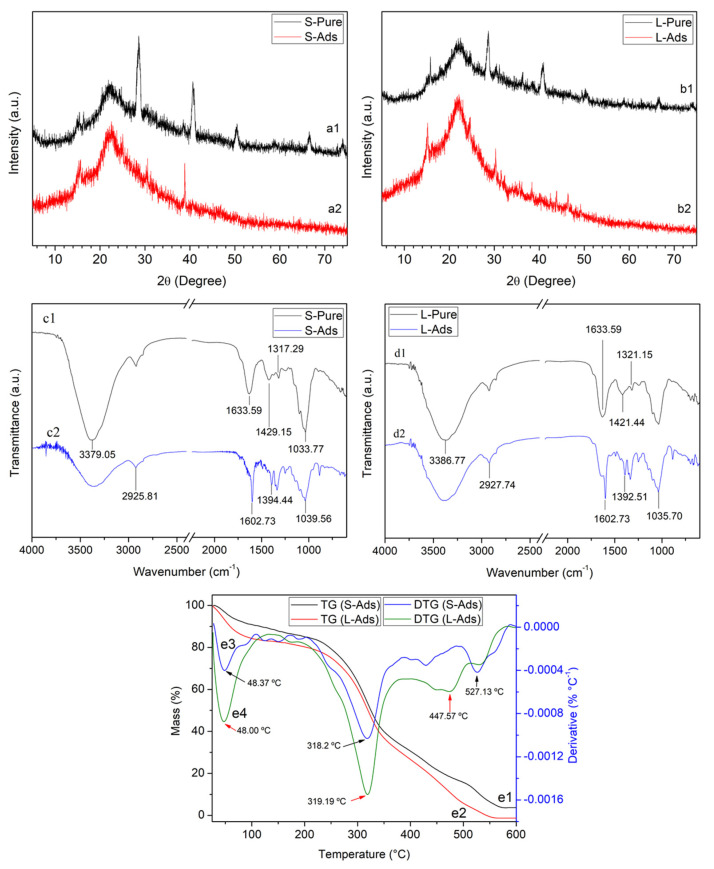
X-ray diffractograms of water hyacinth stem: (a1) before adsorption, (a2) after adsorption; and leaf:(b1) before adsorption, (b2) after adsorption; FTIR of water hyacinth: stem: (c1) before adsorption, (c2) after adsorption; and leaf: (d1) before adsorption, (d2) after adsorption; TG of water hyacinth: (e1) stem and (e2) leaf after adsorption. DTG of water hyacinth: (e3) stem and (e4) leaf after adsorption.

Characterizations of the materials after adsorption were carried out to understand how adsorption affects the studied materials. After adsorption, the XRD results show an increase in amorphous regions, with the disappearance and reduction of intense peaks from 2θ = 40° (Figure 8 (a2 and b2)), both for the stem and leaf material, which may indicate that diffusion of the adsorbate to the internal local sites changed its structure. Furthermore, after adsorption of the cationic dye, the intense peak of the stems decreased compared to that of the leaves. Thus, it can be concluded that the amount adsorbed by the stems is greater than the leaves [73].

Note that after MB adsorption, the intensity also changed in the 3300 cm^−1^ band in both spectra, which may indicate the interaction of adsorbent and adsorbate (Figure 8 (c2 and d2)). This corroborates the XRD results, in which an increase in amorphous regions was observed. This is because this region is completely amorphous, giving access to adsorbed molecules to penetrate the surface of the material [74]. In this context, the negative surface also charges, referring to the O-H phenolic groups, facilitating adsorption by electrostatic attraction with MB, a cationic dye [75]. 

Furthermore, two characteristic bands of MB appear at 1487 and 1604 cm^−1^ representing the vibration of elongation C=C and bond C=N in aromatic rings, respectively [76]. The FTIR spectrum of leaves and stems loaded with MB shows that the bands are displaced from their position, and the intensities change. These results indicate the establishment of interactions between MB dye molecules and the functional groups of leaves and stems [73]. 

Regarding the results of thermal analysis after adsorption, TG and DTG (Figure 8 (e1–e4)) indicate that the thermal profile of the stem and leaf remained in three stages. The first mass loss event with maximum temperature for the S-Pure material of 43 °C (Figure 1 g3) changed to 48 °C after adsorption in S-Ads (Figure 8 (e3)); the second event for S-Pure at 307 °C changed to 318 °C after adsorption in S-Ads; and the third event for S-Pure from 466 °C changed to 527 °C after adsorption in S-Ads.

Similarly, there were some changes in the mass loss events for the L-Pure material. The first event with a maximum temperature of 53 °C (Figure 1 (g4)) changed to 48 °C after adsorption in L-Ads (Figure 8 (e4)); the second event for L-Pure at 301 °C changed to 319 °C after adsorption in S-Ads, and the third event for L-Pure at 506 °C changed to 477 °C after adsorption in L-Ads. 

Thus, this may indicate the interaction of the dye with the water hyacinth, as corroborated by the FTIR and XRD results. Finally, after adsorption, there was a total degradation of leaf biomass at approximately 550 °C and less than 5% residue for the stem at maximum temperature. This can be explained by the alteration of the material structure due to MB adsorption, leaving the structure more amorphous, as shown in XRD, and thus reducing its degradation temperature.

### 3.4. Material Reuse

**Figure 9:**
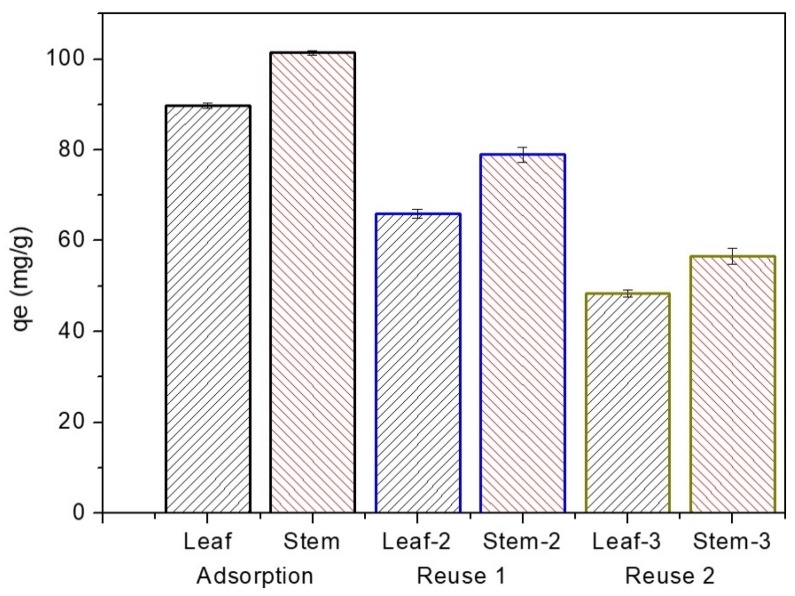
Material adsorption: leaf and stem; reuse 1: leaf-2 and stem-2; reuse 2: leaf-3 and stem-3.

The study of MB desorption with stem and leaf bioadsorbents is important for the material to be reused again in the adsorption process [36,77]. The adsorption, desorption, and reuse experiments (first, second, and third times) were performed and described in the methodology. In Figure 9, adsorption of MB from the stem biomass was 101.42 mg.g^−1^ ± 0.48, and biomass leaf was 89.83 mg.g^−1^ ± 0.60. After desorption, the material was reused for the first time, and the adsorption capacities of stem-2 and leaf-2 were 78.97 mg.g^−1^ ± 0.67 (80%) and 65.98 mg.g^−1^ ± 0.98 (65%), respectively, verifying there is a slight decrease in the adsorption capacity when compared to the previous adsorption. For the second reuse of materials, the adsorption values continued to decrease for stem-3 and leaf-3, being 56.57 mg.g^−1^ ± 1.77 (64%) and 48.43 mg.g^−1^ ± 0.77 (56%), respectively. From the two evaluated cycles, the stem showed higher levels of adsorption than the leaf, but significantly lower retention of the initial levels of adsorption.

Corroborating our research, Guo, Liang, and Tian [78] studied the heavy metal ions removal from aqueous solutions by adsorption using modified orange peel as an adsorbent. The authors observed that after the reuse experiments, the adsorption efficiencies decreased after the first adsorption-desorption cycle, maintaining the adsorption of around 78% and 75% of the materials.

The adsorption capacity of dyes by bioadsorbents decreases slightly after regeneration, as 100% desorption or destruction of adsorbed dyes does not occur during regeneration [79]. However, for the commercial application of water hyacinth, it may not be necessary to regenerate and reuse the biosorbent if the cost of such processes exceeds the cost of replacement. Water hyacinth could be easily substituted since the material is low cost and readily available as biological waste [80]. Furthermore, the remaining ash can be used in landfills and in the production of fire bricks, building blocks, and other building materials [81], thus completing waste recycling.

Despite the efficient use of water hyacinth for the decontamination of pollutants, future research should be carried out with the application of carbonization and activation of the material to extend the reuse of this biomass in several cycles, aiming to combat the deficiency of ineffective removal of pollutants within limited reuse cycles and the use or valorization of the water hyacinth.

### 3.5. Comparative Profile

To evaluate the development behavior of the new adsorbent proposed in this research, a comparison study was performed between the leaf and stem biomass of the water hyacinth and other adsorbents reported in previous studies (Table 4). Notably, water hyacinth biomass has excellent adsorption capacity for MB dye and a fast adsorption process, as it reaches equilibrium in about an hour, standing out in relation to other bioadsorbents found in the recent literature.

## 4. Conclusions and Future Prospects

The results obtained allow us to conclude that the water hyacinth (*Eichhornia crassipes*) biomass has a microcrystalline profile due to the presence of lignin in its constitution, by XRD analysis. The analysis of the FTIR spectra of the water hyacinth parts indicated the presence of characteristic bands of cellulose and lignin. Regarding the morphological structures, the biomass presented irregular structures, thus demonstrating heterogeneity on the surface. The thermogravimetric curves (TG) show that the stem achieved a higher residual content than the leaf, which may be related to hemicellulose, lignin, and mineral components that are present in greater quantity in the stem.

The adsorption data better fit the pseudo-second-order model for the sorption kinetics of MB, with 90.8% and 89.9% removal for the stem and leaf biomass, respectively, in contact time of up to 60 min. The maximum adsorption occurred at natural pH. The equilibrium studies elucidated by the isotherm of both bioadsorbents that the adsorption is favorable and that the data better fit the Langmuir model, indicating active binding sites with the same affinity as MB.

However, in the process of desorption and reuse of the material, with each reuse, the materials gradually decrease the adsorption capacity, with the stem showing higher levels of adsorption in relation to the leaf. 

Nevertheless, it is possible to affirm that the application of the water hyacinth bioadsorbent is a renewable, potentially efficient, economical resource and can be used for the removal of industrial effluents via an adsorption process, with the purpose of also mitigating the aquatic pollution caused by its uncontrolled growth.

## Figures and Tables

**Table 1:** Mean and standard deviation (± SD) of fiber fraction composition (triplicate) of stem and leaf of *Eichhornia crassipes* (water hyacinth).

Fiber Fraction Composition	*Eichhornia crassipes*	
	Stem	Leaf
Dry matter-DM (% as fresh matter)	91.63 ± 0.01	91.36 ± 0.02
Neutral detergent fiber (%DM)	68.91 ± 0.38	58.88 ± 0.91
Acid detergent fiber (%DM)	38.67 ± 1.06	29.17 ± 0.42
Acid detergent lignin (%DM)	10.88 ± 1.40	8.36 ± 0.66
Cellulose (%DM)	27.79 ± 0.20	20.81 ± 0.17
Hemicelluloses (%DM)	30.24 ± 0.32	29.71 ± 0.28

**Table 2:** Constant of the kinetic models of the stem and leaf of water hyacinth (*Eichhornia Crassipes*) in the adsorption of the methylene blue cationic dye.

		Pseudo-First-Order				Pseudo-Second-Order		
Adsorbent	q_e_ (mg.g^−1^)	K_1_ (min^−1^)	Error	R^2^	q_e_ (mg.g^−1^)	K_2_ (mg.g^−1^ min^−1^)	Error	R^2^
**Stem**	06.29	0.0016	0.11028	0.24042	50.42	0.0811	0.00566	0.99996
**Leaf**	11.16	0.0027	0.11048	0.52553	46.88	0.0141	0.00565	0.99996

q_e_ amount of dye adsorbed at equilibrium (mg.g^−1^); K_1_ adsorption constant of the first-order model (min^−1^); K_2_ s-order model constant (mg.g^−1^ min^−1^), and R^2^ linearity coefficient.

**Table 3:** Constant of the isothermal models of the stem and leaf of water hyacinth (*Eichhornia Crassipes*) in the adsorption of the cationic dye methylene blue.

		Langmuir				Freundlich		
Adsorbent	qmax (mg.g^−1^)	KL (mg.L^−1^)	Error	R2	1/n	K_F_ (mg.g*−1*)	Error	R^2^
Stem	153.84	0.024	0.025	0.9935	1.700	6.724	0.236	0.9264
Leaf	153.84	0.017	0.047	0.9848	1.828	6.881	0.210	0.9383

q_max_: maximum amount of species adsorbed per mass of bioadsorbent (mg.g^−1^); 1/n: constant related to surface heterogeneity; K_L_: Langmuir adsorption constant at chemical equilibrium between adsorbate and adsorbent (mg.L^−1^); K_F_: Freundlich adsorption constant related to adsorption capacity and R^2^ linearity coefficient.

**Table 4:** Comparison of the maximum adsorption of methylene blue by the bioadsorbent produced from the leaf and stem of water hyacinth with various adsorbents.

Adsorbent	q_max_ (mg.g^−1^)	References
Cotton gin rubbish dust	112.6	[82]
Corn cob	405.22	[83]
Bread nutshell	409.00	[84]
*Terminalia catappa* (Indian almond) husks	88.62	[85]
White pine sawdust	87	[86]
Soy husk	169.90	[87]
*Punica granatum* bark	10.7296	[88]
Brown algae *Sargassum muticum*	9.55	[89]
Rice straw	20.38	[90]
*Streptomyces fradiae* biomass	59.63	[91]
Pitaya peels	190.30	[92]
Pomegranate peels	200.0	[93]
*Syringa vulgaris* leaf powder	188.2	[94]
Weeds	41,67	[95]
*Cucumis sativus* bark	20.1410	[96]
Walnut shell powder	142.85	[97]
Water hyacinth stem	50.42	This study
Water hyacinth leaf	46.88	This study
